# Global Stakeholder Perspectives on Real-World Data and Evidence in Health Technology Assessment: An Exploratory Study

**DOI:** 10.3390/healthcare14060822

**Published:** 2026-03-23

**Authors:** Konstantinos Zisis, Elpida Pavi, Mary Geitona, Kostas Athanasakis

**Affiliations:** 1 Laboratory for Health Technology Assessment (LabHTA), Department of Public Health Policy, School of Public Health, University of West Attica, 11521 Athens, Greece; 2 Institute for Health Economics, 11521 Athens, Greece; 3 Department of Social & Educational Policy, Faculty of Social and Political Sciences, University of Peloponnese, 20100 Corinth, Greece

**Keywords:** real-world data, real-world evidence, health technology assessment, stakeholder perspectives, data quality, international collaboration

## Abstract

**Objective:** This exploratory study presents an international, multi-stakeholder snapshot of perceptions regarding real-world data and real-world evidence in health technology assessment. The aim is to identify perceived opportunities, barriers, and enabling conditions rather than to generate generalizable conclusions. **Methods:** A 21-item, expert-validated questionnaire was distributed via LimeSurvey to diverse health technology assessment stakeholders, including academia, industry, health technology assessment agencies, healthcare providers, policymakers, patients, and payers. The survey explored perceptions of value, methodological and regulatory challenges, and future outlooks for RWD/RWE use in HTA. Ethical approval was obtained by the University of West Attica Ethics Committee, and pilot testing was conducted prior to dissemination. Data were analyzed using descriptive statistics, consistent with the study’s exploratory intent and acknowledging that results are preliminary and not statistically generalizable. **Results:** Thirty-two completed responses demonstrated preliminary stakeholder support for integrating real-world data and real-world evidence into health technology assessment. Respondents represented academia, industry, HTA agencies, healthcare providers, policymakers, and patient/advocacy groups; however, no payer responses were obtained. Respondents emphasized the value of real-world data in complementing clinical trials by capturing real-world effectiveness, patient diversity, and long-term outcomes, especially in rare diseases and cancer. Key challenges included poor data quality, confounding biases, and regulatory barriers. Stakeholders highlighted the importance of standardization, transparency, and international collaboration. Opportunities included better decision-making, personalized healthcare, and improved post-market monitoring, with strong calls for robust infrastructure, clear methodologies, patient involvement, and supportive health policy frameworks. **Conclusions:** Real-world data and evidence enhance health technology assessment by supporting better decisions and personalized care. However, issues like data quality, methods, and trust must be addressed through standardization, strong infrastructure, and collaboration to ensure effective and impactful implementation in healthcare, while acknowledging these insights are based on a small exploratory sample.

## 1. Introduction

The integration of Real-World Data (RWD) and Real-World Evidence (RWE) into Health Technology Assessment (HTA) represents a transformative shift in how healthcare interventions are evaluated and implemented, reflecting a broader evolution in evidence-based decision-making beyond the traditional hierarchy dominated by randomized controlled trials (RCTs). Integrating RWD/RWE into HTA offers multiple benefits, improving insights into treatment effectiveness, resource use, and cost analysis, especially using patient registries [1]. Several countries have started integrating RWD/RWE into HTA and coverage decisions. For example, the U.S. Food and Drug Administration’s (FDA) Real-World Evidence Program has informed regulatory approvals [2], and the National Institute for Health and Care Excellence (NICE) in the United Kingdom has used RWD to guide coverage for innovative therapies [3]. Traditional RCTs, while robust in design, face several limitations; one of which is their limited generalizability in capturing the complexities of diverse patient populations and real-world clinical settings. This occurs due to selection bias—when the participants chosen for the study do not accurately represent the general population—or due to specific characteristics of the study population that may not be relevant to others [4].

RWD, defined as health-related data derived from various sources such as electronic health records (EHRs), patient registries, claims and billing activities, patient-generated data (e.g., patient-reported outcomes) and even digital health tools, offers a comprehensive view of patient outcomes and healthcare utilization. Real-world data from these sources can be gathered and analyzed using various study designs, including prospective and retrospective cohort studies, case–control studies, and pragmatic clinical trials [5]. When analyzed to produce RWE, these data provide valuable insights into the effectiveness, safety, and value of health technologies outside the controlled environment of clinical trials. Within HTA decision-science, RWD and RWE are increasingly positioned as complementary forms of evidence that support lifecycle and deliberative assessment models rather than replacing RCTs outright. The integration of RWD and RWE into HTA processes has the potential help address uncertainties, reduce healthcare costs, and enhance patient and societal benefits [6].

Despite the clear advantages, the adoption of RWD and RWE in HTA is met with several challenges, including issues related to data quality, standardization, regulatory acceptance, and methodological approaches. These challenges are not solely technical; they are shaped by institutional trust, regulatory risk aversion, and established epistemic preferences for controlled trial evidence within HTA systems. Various stakeholders are involved in these processes: regulators evaluate evidence and ensure safety and efficacy; payers assess cost-effectiveness and reimbursement decisions; clinicians and providers contribute real-world practice insights; patients and advocacy groups provide experience-based outcomes; and industry supports evidence generation and implementation. Understanding these challenges and particularly the perspectives of various stakeholders is crucial for the successful integration of RWD and RWE into HTA frameworks.

Accordingly, this study is intentionally exploratory in nature and seeks to provide a preliminary, stakeholder-informed snapshot rather than generalizable conclusions. It aims to explore the views of international stakeholders across different roles on the utilization of RWD and RWE in HTA. By capturing insights from a diverse group of participants, including academics and researchers, industry professionals, policymakers, and patient advocates, the study aims to map areas of alignment, perceived barriers, and aspirational opportunities in RWD/RWE use, while acknowledging that deeper causal explanations and stakeholder trade-offs require subsequent qualitative and quantitative investigation. The study thereby contributes to understanding how international perspectives can inform HTA practice and guide policy decisions, offering practical implications for improving the integration of RWD/RWE in global healthcare decision-making and HTA, as well as shaping health policies.

## 2. Materials and Methods

### 2.1. Data Collection Methods and Questionnaire Overview

Data were collected through an electronic questionnaire disseminated via the LimeSurvey tool, enabling efficient acquisition of insights from international HTA stakeholders. The study was designed as an exploratory, cross-sectional survey intended to capture a preliminary snapshot of stakeholder perceptions rather than to generate generalizable or comparative estimates. The survey comprised 21 carefully selected questions, structured into key sections addressing different aspects of RWD acceptance and utilization in HTA. The design prioritized clarity and accessibility, considering the diverse international audience, including HTA agencies, payers, industry representatives, researchers, and patient advocates, and recognizing that the sample size would not allow for formal subgroup comparisons across stakeholder categories. An informed consent agreement was presented on the first page to ensure voluntary participation. The first section gathered demographic information to contextualize responses, helping to analyze variations based on participants’ backgrounds. The second section explored stakeholders’ perspectives on the role and value of RWD and RWE in HTA. This included their perceived importance, applicability across health technologies and therapeutic areas, and preferences for optimal RWD sources. The third section examined barriers to integrating RWD and RWE into HTA, identifying challenges that may hinder their effective use in decision-making processes. Lastly, the final section focused on anticipated developments, benefits, and opportunities associated with RWD/RWE integration, offering insights into how stakeholders envision its future role in HTA and key areas for improvement.

The questionnaire underwent validation and reliability testing following Kimberlin and Winterstein (2008) [7]. In addition, an expert panel of four representatives from the academic environment assessed content and face validity. Pilot testing involved health economics, policy, and HTA experts, with feedback incorporated into the final version. The full questionnaire is provided as Supplementary Materials to allow readers to assess the wording of questions, response options, and potential sources of response framing.

Ethics approval was granted by the University of West Attica (approval number: 24994—28 March 2024). Dissemination occurred from May to July 2024 via LimeSurvey, direct email channels, and LinkedIn. Stakeholders were identified through web searches, LinkedIn, and international organizations, including the Professional Society for Health Economics and Outcomes Research (ISPOR). Eligible participants were systematically selected to ensure accurate role validation and updated contact information. Given the dissemination period and recruitment channels, participation may reflect engagement within specific professional networks active during the study timeframe, which is acknowledged as a potential source of sampling bias.

To minimize non-response bias, a single reminder was sent to participants, and recipients were encouraged to forward the survey to a more appropriate respondent if needed. All responses were collected anonymously in compliance with General Data Protection Regulation (GDPR) regulations. Data analysis was conducted in Microsoft Excel 2016 using descriptive statistics for visualization, consistent with the exploratory aim of summarizing patterns of perception rather than testing hypotheses or conducting inferential analyses. Comparative or stratified analyses were intentionally not conducted due to sample size constraints.

### 2.2. Stakeholders’ Population Determination

Participants were drawn from diverse stakeholder groups to ensure a comprehensive perspective on the use of RWD and RWE in HTA. The inclusion of multiple stakeholder categories was intended to capture breadth of perspectives rather than proportional representativeness. The stakeholder categories included:

Academia: Researchers, scholars, and institutions focused on HTA, health services research, and policy.

Industry: Representatives from pharmaceutical, medical device, and consulting companies.

HTA Agencies: Experts involved in evidence assessment.

Healthcare Providers: Professionals across hospitals and health systems.

Policymakers and Scientific Organizations: Decision-makers in healthcare policy.

Patients and Advocacy Groups: Individuals and representatives with firsthand health technology experience.

Payers: Insurance and government healthcare funders.

These stakeholder categories were selected to reflect the main actors involved in HTA, including evidence assessment (HTA agencies), clinical implementation (healthcare providers), reimbursement decisions (payers), evidence generation (academia and industry), policy development (policymakers), and patient-centered input (patients and advocacy groups).

### 2.3. Sampling Method

A structured invitation-based recruitment strategy was used to reach diverse stakeholder groups involved in HTA, segmented by specific characteristics that reflect their roles within the study. While stakeholder categories were identified in advance to guide outreach efforts, participation ultimately relied on voluntary response and should not be interpreted as statistically representative of the global HTA community nor as reflecting proportional distribution across stakeholder groups. The sample was determined through the following steps:

(1) Identification of Strata: Participants were categorized into distinct strata based on two primary criteria:
  - Affiliation: Stakeholders were grouped by their professional role or organizational type, such as healthcare providers, industry representatives, policymakers, and patient advocacy groups.
  - Location: Participants were further stratified geographically to guide outreach across various regions and settings. However, final participation depended on voluntary response and may reflect over- or under-representation of certain subgroups.
(2) Population Estimation: The target population was estimated through a comprehensive multi-source identification strategy. Eligible participants were identified using:
  - Databases and Professional Directories: Industry registries, health technology assessment (HTA) stakeholder directories, and academic databases were reviewed to compile lists of potential participants.
  - Organizational Sources: Professional organizations, research institutions, and healthcare networks were contacted to identify eligible stakeholders within their networks.
  - LinkedIn Searches: Direct searches through LinkedIn allowed for the identification of professionals with relevant expertise and affiliation, ensuring up-to-date contact information and role validation.
(3) Planned Outreach Distribution: Invitation targets were distributed across stakeholder categories based on estimated prevalence within the HTA ecosystem; however, the achieved sample did not reflect proportional representation due to differential response rates.
(4) Participant Identification and Invitation: Approximately 30 individuals per stakeholder category were identified through professional directories, organizational listings, academic databases, and LinkedIn searches, with an overall invitation target of 150 individuals. Invitations were distributed systematically across categories; however, participation was voluntary and subject to self-selection.
(5) Sample Size Determination: The target sample size was based on feasibility and expected response rates and was considered appropriate for an exploratory study. The achieved sample size does not permit statistically powered subgroup analyses and are therefore interpreted descriptively and qualitatively rather than as generalizable estimates.

## 3. Findings

**Figure 1:**
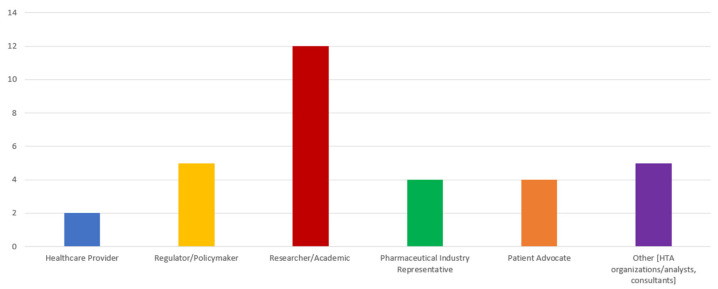
Respondents’ role within the healthcare system.

The survey received 32 completed questionnaires (21.3% response rate) from respondents across all global regions, except Africa. Not all participants responded to every question, which is acknowledged as a limitation and discussed in detail later in the manuscript. Researchers/academics comprised the largest group (12/32, 37%), highlighting their strong involvement in HTA and RWD/RWE discussions. Regulators/policymakers accounted for (5/32, 16%), followed by patient advocates (4/32, 13%), pharmaceutical industry representatives (4/32, 12%), and HTA/consulting professionals (5/32, 16%). Healthcare providers were the least represented group (2/32, 6%) (Figure 1).

### 3.1. Perceptions of RWD for HTA

Stakeholders across various healthcare sectors are increasingly recognizing the significance of integrating RWD and RWE into HTA frameworks. Table 1 provides findings, with responses from diverse stakeholders, showing broad support for the integration of RWD into HTA. When respondents were asked about the importance of RWD in HTA in their country, the majority (20/32, 62.5%) identified key benefits such as improved decision-making, increased relevance to patient populations, and the ability to assess long-term outcomes. Across different roles—government agencies, researchers, market access directors, and patient advocates—there is a shared consensus that RWD complements RCTs. While RCTs provide controlled efficacy data, RWD offers valuable insights into real-world effectiveness, patient outcomes, and long-term safety. Patient advocates, in particular, underscored the importance of RWD in capturing heterogeneous and often underrepresented patient populations, thereby addressing limitations inherent in traditional clinical research. This perspective was echoed by respondents from HTA bodies, academia, and industry, all of whom recognized RWD’s potential to fill critical evidence gaps—especially in areas related to treatment adherence, real-world utilization, and longitudinal impact.

**Figure 2:**
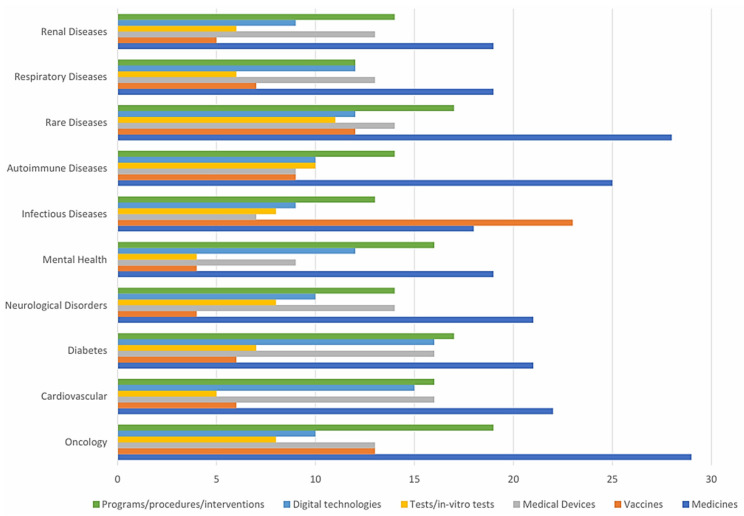
Key health technologies and disease areas for RWD and RWE application in HTA.

Identifying which diseases benefit most from RWD and RWE is crucial (Figure 2). The survey responses suggest that rare diseases and oncology derive the most benefit from RWD, reflecting their complex, evolving nature and high unmet medical needs. Additionally, the application of RWD in oncology, infectious diseases, and neurological disorders highlights the continued dominance of pharmaceutical evaluations in HTA.

**Figure 3:**
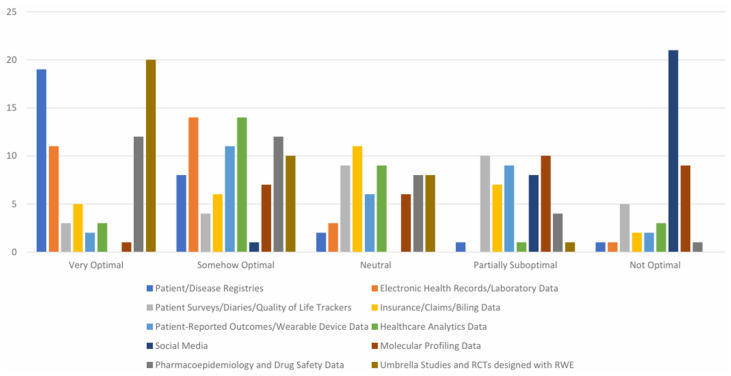
Optimal Real-World Data types for HTA evaluations.

Figure 3 presents a comparative assessment of the perceived optimality of various RWD sources for generating RWE. Patient and disease registries were most frequently rated as “Very Optimal” (19 respondents), suggesting high confidence in their validity and reliability for RWE generation. EHRs/laboratory data (11 respondents) and umbrella studies or RCTs designed with RWE principles (20 respondents) also received favorable evaluations. In contrast, social media was predominantly rated as “Not Optimal” (21 respondents), indicating limited perceived utility due to potential concerns regarding data quality, representativeness, and methodological rigor. Other RWD sources, including insurance/claims data (5 respondents rated “Very Optimal” and 11 “Neutral”), molecular profiling data (10 respondents rated “Partially Suboptimal” and 9 “Not Optimal”), pharmacoepidemiology and drug safety data (12 respondents rated “Very Optimal”), and patient-reported outcomes/wearable device data (11 respondents rated “Somehow Optimal”), elicited more varied responses across categories. These findings underscore the importance of carefully matching data sources to research objectives and highlight ongoing uncertainty regarding the utility of less conventional RWD sources.

### 3.2. Barriers and Challenges of RWD/RWE Inclusion Within the HTA Process

**Figure 4:**
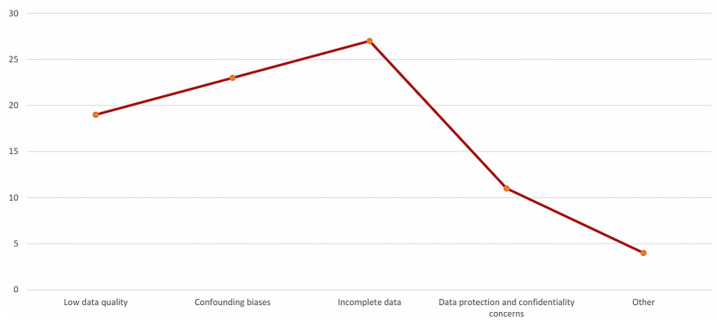
Main challenges encountered in the quality and reliability of RWD in HTA.

The challenges associated with the use of RWD and RWE in HTA are multifaceted (Figure 4).

These challenges encompass data quality issues, methodological limitations, regulatory constraints, and stakeholder acceptance, all of which influence the integration of RWD/RWE into HTA processes. Among the most frequently reported challenges are incomplete data, reported by 27 respondents (84%), which limits the comprehensiveness and accuracy of RWD analyses. Many stakeholders struggle with datasets that fail to capture all necessary patient information, leading to gaps in evidence. Confounding biases also emerged as a major concern, identified by 23 respondents (72%), emphasizing the difficulty in ensuring that real-world findings are not distorted by external factors. Low data quality further compounds these issues, cited by 19 respondents (59%), as inconsistencies and errors in data collection and processing affect reliability. Additionally, concerns surrounding data protection and confidentiality, although less frequently cited, were reported by 11 respondents (34%), and remain significant regulatory and ethical barriers that restrict data sharing and access. While other minor challenges were mentioned, they appeared less frequently or varied across stakeholder groups, with four respondents (13%) selecting “Other”.

**Figure 5:**
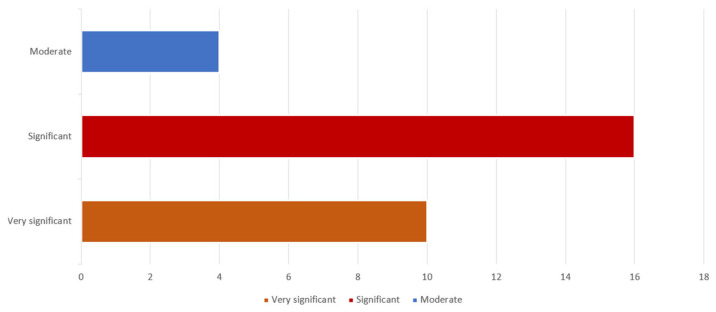
Perceived significance of methodological challenges in HTA.

In addition to data quality concerns, methodological challenges were identified as highly significant in HTA applications (Figure 5). 

A substantial portion of respondents categorized these challenges as “significant,” with 16 respondents (53%) selecting this option, indicating their critical impact on the reliability of RWD/RWE in HTA decision-making. Another large segment classified them as “very significant,” with 10 respondents (30%), underscoring the urgency of addressing these issues to maintain the credibility of HTAs. A smaller portion of respondents viewed these challenges as "moderate," with four respondents (17%), suggesting that while relevant, they are not universally regarded as highly detrimental. 

**Figure 6:**
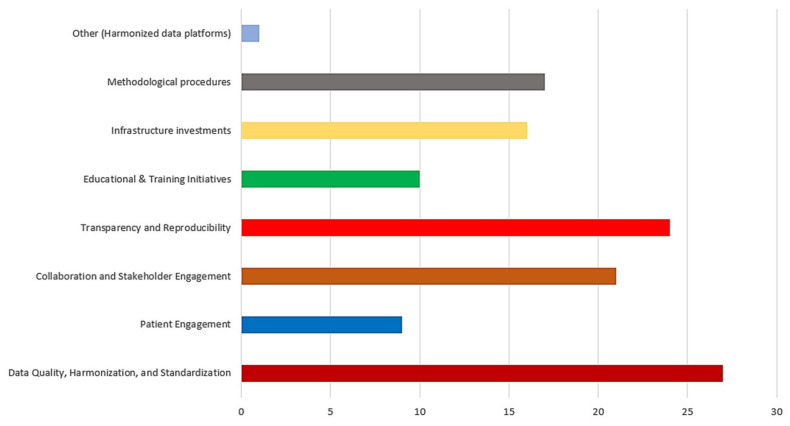
Strategies for overcoming barriers in utilizing RWD-RWE for HTA.

Beyond data quality and methodological concerns, respondents also highlighted broader challenges influencing the adoption and acceptance of RWD and RWE, summarized in Table 2. These include unclear definitions and objectives, biases in data collection by HTA bodies, and payer skepticism regarding RWD reliability, which could lead to demands for larger discounts on health technologies. Stakeholder acceptance is particularly crucial in fields like oncology, where collaboration between different entities is essential. Other critical concerns include limited transparency in data sharing, restricted access to high-quality databases, and inconsistencies in international methodologies. Political knowledge gaps, industry-funded registry biases, and the need for independent analysts further complicate the use of RWD in HTA. To address these challenges, respondents proposed multi-faceted solutions (Figure 6).

Standardizing data collection through uniform definitions and formats was a key recommendation to enhance reliability and comparability across datasets, identified by 27 respondents under data quality, harmonization, and standardization priorities. Greater patient engagement was emphasized to ensure that HTA reflects real-world patient experiences and needs, selected by nine respondents. The establishment of clear guidelines and robust methodologies was highlighted as necessary to mitigate the risks associated with low-quality evidence, with 17 respondents prioritizing methodological procedures. Implementing common data models to facilitate cross-dataset comparisons was another widely endorsed strategy, reflected in 24 respondents emphasizing transparency and reproducibility. Moreover, fostering collaboration between patients, healthcare providers, regulators, and researchers is crucial for strengthening the credibility and acceptance of RWD in HTA, with 21 respondents highlighting collaboration and stakeholder engagement. Capacity building was also identified as an important enabler, with 10 respondents **(31%)** emphasizing educational and training initiatives to improve methodological competencies and appropriate RWD use. In addition, 16 respondents (50%) highlighted the need for infrastructure investments to support high-quality data generation and sharing. Harmonizing HTA guidelines across agencies and streamlining methodologies to address confounding biases and incomplete data were seen as vital steps toward optimizing RWD integration, supported by 17 respondents emphasizing methodological harmonization. Lastly, respondents stressed the need for international agreements on standardized platforms to support prospective RWD collection, with one respondent selecting other harmonized data platform initiatives, ensuring that HTA frameworks are equipped to leverage real-world evidence effectively.

### 3.3. Future Outlook and Opportunities

Building on the identified challenges, forward-looking opportunities and strategic actions were explored for optimizing the integration of RWD and RWE into HTA. Stakeholder insights reveal critical areas for improvement, including collaborative efforts, policy development, and infrastructure enhancement to support more responsive, patient-centered, and evidence-informed decision-making in healthcare.

#### 3.3.1. Opportunities and Collaborative Efforts for Utilizing RWD and RWE in HTA: Stakeholder Insights and Prioritization

Respondents highlighted several opportunities for leveraging RWD and RWE to enhance HTA, spanning evidence improvement, patient perspectives, and comparative effectiveness research.

**Figure 7:**
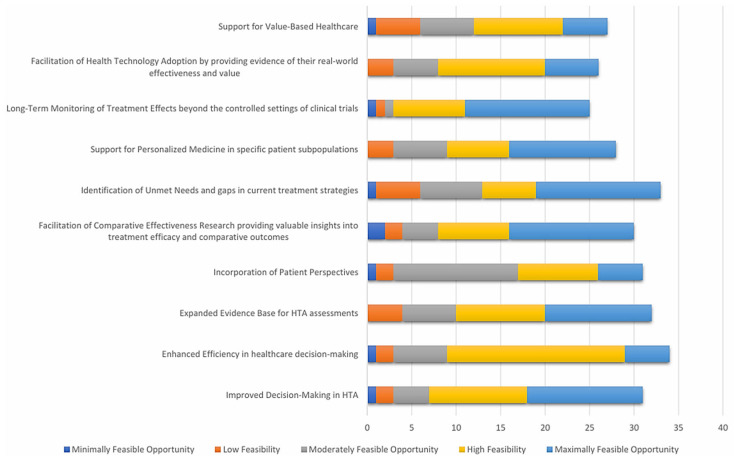
Key opportunities for integrating RWD and RWE in HTA.

Figure 7 illustrates stakeholder assessments of the feasibility of and opportunity for applying RWE across key domains of HTA. The majority of respondents rated domains such as ‘’Improved Decision-Making in HTA’’ (30 respondents rated High or Maximal Feasibility), ‘’Enhanced Efficiency in Healthcare Decision-Making’’ (32 respondents rated High or Maximal Feasibility), and ‘’Expansion of the Evidence Base’’ (30 respondents rated High or Maximal Feasibility) as having high to maximal feasibility. Conversely, areas like ‘’Support for Value-Based Healthcare’’ (27 respondents rated Moderate-to-High Feasibility) and ‘’Facilitation of Technology Adoption’’ (26 respondents rated Moderate-to-High Feasibility) showed a greater proportion of moderate or low feasibility responses. These findings suggest broad support for the integration of RWE into HTA processes, particularly in domains directly linked to evidence synthesis, patient-centered evaluation, and long-term outcomes.

**Figure 8:**
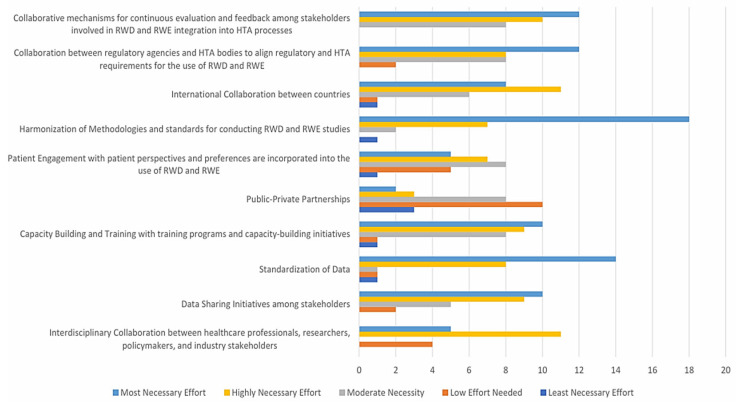
Collaborative endeavors to enhance the integration of RWD and RWE into HTA.

On the other hand, Figure 8 presents the stakeholder prioritization of efforts needed to support the integration of RWD and RWE into HTA processes. The highest priority was assigned to harmonization of methodologies (18 respondents), followed by standardization of data (14 respondents), international collaboration between countries (12 respondents), and collaboration between regulatory agencies and HTA bodies (12 respondents). These areas were predominantly rated as requiring the “Most Necessary Effort”. These findings highlight the perceived need for coordinated global action, regulatory alignment, and methodological consistency to enable robust and scalable RWE implementation in HTA.

#### 3.3.2. Essential Policy Measures for Integrating RWD and RWE into HTA: Insights from Stakeholder Perspectives

To ensure the effective integration of RWD and RWE into HTA, targeted policy measures are essential. Stakeholder feedback underscores the need for structural, regulatory, and methodological reforms to address current limitations and variability in the generation and application of RWE. Building on the prioritization of efforts identified in Figure 8, this section synthesizes key policy directions that are deemed critical for facilitating the robust and consistent use of RWD and RWE across HTA frameworks (Table 3).

Survey findings highlight five critical areas: standardization, transparency, international collaboration, patient engagement, and infrastructure development. Standardization is essential for ensuring data consistency across studies, with respondents emphasizing the need for uniform methodologies. However, overly rigid standards may limit innovation, necessitating a balanced approach that allows methodological flexibility. Transparency is crucial for reproducibility and trust, requiring clear documentation of data sources, analytical methods, and assumptions to ensure scientific integrity. International collaboration is seen as a way to address data fragmentation and improve the generalizability of HTA findings. However, regulatory and logistical challenges must be addressed to facilitate data sharing across regions. Patient engagement is critical to ensuring RWD reflects patient perspectives, aligning with the shift toward patient-centered care. Involving patients in study design can lead to more relevant and impactful outcomes. Infrastructure development is necessary for effective RWD integration, requiring investment in data-capture technologies, personnel training, and centralized coordination. Without strong infrastructure, the benefits of RWD and RWE may remain underutilized.

## 4. Discussion

This study highlights the varying degrees of acceptance and utilization of RWD and RWE across HTA processes globally, emphasizing both the potential and the challenges these data sources present. It highlights both the promising opportunities and the persistent barriers to the successful adoption of RWD/RWE in HTA, drawing upon insights from a diverse range of stakeholders. Findings may reflect perceived or aspirational practices rather than documented implementation. From a conceptual perspective, the observed patterns can be understood through frameworks of innovation adoption, HTA decision science, and epistemic hierarchies, which help explain why some stakeholders embrace RWD/RWE more readily than others and why barriers persist. For example, regulators’ preference for certainty and payers’ risk aversion reflect institutional and epistemic factors that influence adoption.

The results demonstrate broad agreement on the potential benefits of RWD/RWE, particularly in improving decision-making efficiency, enhancing personalized medicine, and expanding the evidence base for HTA. However, significant challenges remain, including data quality issues, methodological inconsistencies, and resistance from stakeholders, particularly payers, who may demand higher discounts due to perceived uncertainty in RWD —reflecting the tension between the industry’s desire for speed and the regulators’ need for certainty, as well as financial risk considerations in reimbursement decisions. It should be noted, however, that no payer representatives participated in the achieved sample; therefore, perspectives related to cost containment, budget impact, and financial sustainability are not directly represented in the present findings and may be underemphasized, and consequently, economic considerations surrounding affordability and reimbursement may have been underestimated in the interpretation of stakeholder priorities.

Our findings align with and expand upon the existing literature. Many studies have highlighted the critical role of RWD/RWE in supplementing traditional clinical trial data to provide more comprehensive, timely, and patient-centered evidence for HTA. As noted by Vaghela S et al. 2024 [8], RWD plays a significant role in the drug approval process for rare diseases and can help bridge gaps in evidence in those diseases, where clinical trials often fall short and RWE might be more relevant and can provide earlier access to relevant treatments, highlight practical issues or clinical gaps that randomized trials might miss, and help identify which patient populations benefit most from specific treatment [9]. In addition, our study findings indicate the importance of patient registries and electronic health records, which have been cited as valuable sources of RWD in the HTA literature [10]. These findings can also be interpreted through decision-science frameworks, which suggest that evidence credibility, relevance, and stakeholder trust are critical for adoption.

The study’s emphasis on patient-centered data aligns with the broader trend in healthcare decision-making that prioritizes patient-relevant outcomes. The integration of patient experience data is essential for developing patient-centered healthcare systems and improving decision-making in regulatory and HTA contexts [11]. This reflects the growing recognition of the value of systematic and meaningful patient engagement in the HTA process, which enhances decision-making by incorporating the perspectives and experiences of those most directly impacted by health technologies [12]. Despite these opportunities, several barriers identified in this study mirror the challenges discussed in the literature, especially concerns about data quality [13], transparency, and the lack of standardized methodologies [14]. These challenges persist not only because of technical limitations but also due to structural and institutional factors, including fragmented data systems, epistemic hierarchies favoring RCTs, and conflicting stakeholder priorities.

However, this study also adds nuance by suggesting specific strategies for overcoming these barriers. While the literature often calls for more collaboration and data-sharing initiatives [15], this research provides actionable recommendations such as the need for harmonized data collection standards, robust transparency practices, and the integration of patient perspectives into HTA processes. By framing these recommendations within systemic and structural considerations, they address both methodological and institutional barriers, thereby facilitating broader adoption and trust in RWD/RWE.

This study has several limitations, common in similar research. First, no payer representatives responded to the surveys, despite their key role in HTA. This absence limits insights into how RWD/RWE adoption might impact system sustainability and resource use. Unfortunately, none of these stakeholders responded to our survey. This limitation also underscores the potential for incomplete representation of conflicting stakeholder priorities. Given the central role of payers in reimbursement and budget allocation decisions, economic and affordability concerns related to RWD/RWE integration may have been underestimated in this exploratory sample, and the balance of perspectives presented may therefore lean toward evidentiary and clinical considerations rather than fiscal constraints. While the sampling strategy targeted 150 participants, the final analyzed sample consisted of 32 respondents due to non-response; therefore, the study is interpreted as exploratory rather than representative. Second, the study relies on self-reported survey data, which may be biased by personal or institutional perspectives, potentially skewing the findings. Third, while aiming for a global view, regional and sectoral differences may not be fully captured. For example, healthcare systems and stakeholder priorities can vary significantly between regions like Europe, North America, and Asia, and between public and private sectors. Additionally, the exploratory nature of the study and the small sample size (n = 32) mean that results should be interpreted as preliminary insights rather than generalizable conclusions.

Despite limitations, this study offers key strengths that enhance its relevance. First, it captures diverse stakeholder views—including HTA agencies, providers, patients, and policymakers—offering a well-rounded look at the challenges and opportunities in using RWD/RWE. However, the absence of payer participation should be considered when interpreting the balance of perspectives presented. Second, the study provides clear, actionable recommendations, offering a practical guide for improving RWD/RWE use in HTA. While valuable, these steps may face barriers like uneven readiness and limited resources, warranting further exploration. Finally, the study’s global lens highlights the need for international collaboration and aligned standards, helping bridge regional gaps and support coordinated healthcare decisions. The findings also suggest that addressing RWD/RWE adoption requires attention to both technical factors (data quality, methodology) and social factors (trust, stakeholder alignment, policy harmonization).

Looking ahead, integrating RWD/RWE into HTA requires standardized methods, transparent processes, and strong data infrastructure. The findings highlight the need for international collaboration and patient engagement to build a resilient, inclusive HTA framework. Flexible yet rigorous policies and clear guidelines are essential to ensure data reliability and usability. Future research should explore specific cases of successful RWD/RWE integration, quantify stakeholder trade-offs, and develop structured frameworks to guide adoption in diverse HTA contexts. These measures can enhance trust, support evidence-based decisions, and promote sustainable, patient-centered healthcare in the evolving landscape of real-world data.

## 5. Conclusions

This study highlights the potential of RWD and RWE to enhance HTA by improving decision-making, supporting personalized medicine, and expanding the evidence base. While stakeholders recognize these benefits, challenges such as data quality concerns, methodological inconsistencies, and payer skepticism remain significant barriers. The findings emphasize the need for standardization, transparency, and international collaboration to ensure the reliable integration of RWD/RWE into HTA processes.

## Figures and Tables

**Table 1:** Key reasons for the importance of including RWD and RWE in HTA.

Respondents	Role Within Healthcare System	Response
Respondent 1	Government agency conducting HTA as advisory role to policy/health care implementation	‘’Real-world data—(particularly within existing published studies in the form of observational or cluster RCTs)—is imperative to understanding some outcomes that are more long term, as well as the improve the understanding of patient adherence and implementation implications from the system perspectives’’.
Respondent 2	Fellow in health economics	‘’To determine the real-world effectiveness and value of health technologies beyond trials’’.
Respondent 3	Representative of a patient association	‘’As a patient advocate, I believe Real-World Data (RWD) is crucial for Health Technology Assessment (HTA) in our country. RWD provides insights into how treatments perform in everyday settings, beyond controlled clinical trials. This data reflects the diverse experiences of patients, including those with multiple health conditions or those not represented in clinical trials. By incorporating RWD, HTA can make more informed decisions that truly reflect patient outcomes, improving the relevance and effectiveness of healthcare interventions. Ultimately, this leads to better healthcare policies and treatments that address the real needs and conditions of patients like myself’’.
Respondent 4	Market access director	‘’PIII studies often lack the “depth of explanation” of the asset studied, as duration of RCTs and the likes is often not more than 1 year. Therefore, RWD is a crucial complementary piece of information for companies to better substantiate their claims and payers/regulators to take more informed decisions. Ultimately in the interest of all parties involved, especially the patients’’.
Respondent 5	HTA analyst for the Peruvian health care system	‘’Sometimes there are evidence gaps to answer clinical question. RWE could he useful in some cases’’.
Respondent 6	HTA analyst	‘’Able to answer HTA questions that other types of data don’t address as well’’.
Respondent 7	Market access and pricing	‘’Provide clinical, economical and impact of a technology on real settings beyond the clinical studies’’.
Respondent 8	Head of pharmacy department—university hospital	‘’Provide a more comprehensive understanding of how medical interventions perform in real-world settings, beyond the controlled environment of clinical trials. This inclusion allows for better-informed decision-making by healthcare stakeholders regarding the effectiveness, safety, and value of healthcare interventions in actual practice’’.
Respondent 9	Previous role between 2000 and 2023 (not indicated)	‘’Real world data reflects the effectiveness of the therapy in patients that resemble the actual patients that will be treated with the therapy’’.
Respondent 10	Not indicated	‘’HTA has been using RWD/RWE for quite a while, what is novel is its use to inform effectiveness evidence (previously only populated by RCT data). In the last decade, particularly in rare diseases and oncology, there has been an increase in reliance of observational data’’.
Respondent 11	HTA RWE manager	‘’Used to contextualise clinical trial results and provide more meaning to them in real-life settings’’.
Respondent 12	Research fellow	‘’RWD/RWE help to understand how a new technology might be used in the population, how different the population might be from the trial population, what treatments are currently used and in what proportions, giving insight into which treatments might be the best comparators’’
Respondent 13	Not indicated	‘’We’ve seen that Italy registries system through AIFA is supporting real-world effectiveness, safety, efficacy data’’.
Respondent 14	Assistant Professor of HTA	‘’Necessity for decision making in absence direct, generalisable RCT evidence. Also valuable for characterisation’’.
Respondent 15	Clinical assessor	‘’Clinical trials rarely answer the most relevant questions from a HTA perspective. Plus, being designed as experiments to isolate efficacy and safety to inform B/R, the main principle is to remove possible confounders, limiting the generalisability of the results to the RW population, which instead is the main concern of HTA evaluations’’.
Respondent 16	Health policy researcher at a university in the UK	‘’RWD and RWE are important for HTA in my country as they provide comprehensive insights into the real-world effectiveness and safety of health technologies. These data sources complement RCTs by reflecting diverse patient populations and everyday clinical practice, ultimately leading to more informed and patient-centered healthcare decisions. UK is already seeing RWE usage in HTA submissions’’.
Respondent 17	Not indicated	‘’Policy makers claim they will use tools like “conditional listing” to improve access; the decision after 2 years will most likely to rely on RWE’’.
Respondent 18	Medical research consultant	‘’Setting the scene in terms of current burden of the disease (epidemilogy, clinical burden economic burden), identify unmet need in current standard of care, provide input for Health Economic modelling’’.
Respondent 19	HTA agency	‘’To compare evidence from pivotal trials to real world evidence’’.
Respondent 20	HTA scientific advisor	‘’It is necessary to conduct good quality HTA’’.

**Table 2:** Challenges influencing the adoption and acceptance of RWD and RWE.

Challenge Category	% (n) *	Representative Comments
Low data quality	72%, 23 respondents	- “Data are collected for other reasons and with another purpose than HTA.” - “Collection of RWD needs to be carefully planned prospectively.”
Confounding biases	76% 24 respondents	- “Important variables are not always collected for confounder adjustment.” - “Usually use claims data or not carefully designed registries.”
Incomplete/missing data	76% 24 respondents	- “High degrees of missing data or variables completely missing.” - “Not all clinicians fill in all fields in registries.”
Data protection and confidentiality concerns	36% 12 respondents	- “Very difficult to access.” - “The current legal framework might need to be optimised.”
Other challenges	40% 13 respondents	- “Lack of patient-relevant data like quality of life.” - “Methodological challenges including limited generalizability.” - “Uncertain source and governance.”

* (based on 25 responses to relevant question).

**Table 3:** Suggested policy and regulatory measures to support integration of RWD/RWE in HTA.

Theme	Illustrative Responses
Data Standardization & Sharing	“Data standardization”, “Guidelines in RWE generation”, “Standards and transparency”, “Standardization of data collection and reporting”
Transparency	“All things related to transparency”, “Specifically point out the weakness of the evidence when they made the regulation decision”, “Transparency and reproducibility”
Patient Involvement	“Patient Advisory Boards or Panels”, “Include patients in HTA committees”, “Patients records should be centralized”
International & Stakeholder Collaboration	“International collaboration”, “Collaboration between all stakeholders”, “European Health Data Space is very exciting”
Infrastructure & Investment	“Public investments on data-capture infrastructures”, “Ensure that privacy rules allow linking”
Critical Reflection & Caution	“Frankly, I don’t believe RWD can play a key role in initial HTA”, “No need to expand further”, “We need a chance to learn by doing”

## Data Availability

The original contributions presented in this study are included in the article/Supplementary Materials. Further inquiries can be directed to the corresponding authors.

## Supplementary Materials

The following supporting information can be downloaded at: https://www.mdpi.com/article/10.3390/healthcare14060822/s1, File S1: Full questionnaire.
