# Supplementary material for: Global Stakeholder Perspectives on Real-World Data and Evidence in Health Technology Assessment: An Exploratory Study
Source: Healthcare (Basel). 2026 Mar 23;14(6):822. doi: 10.3390/healthcare14060822 (PMC13027187; doi:10.3390/healthcare14060822)
Supplement: Supplementary file 1 [file healthcare-14-00822-s001.zip › healthcare-4046469-supplementary.pdf]

...

Change language English - English

Stakeholder Perspectives on Real-World Data and Real-World Evidence for Health Technology Assessment

There are 21 questions in this survey.

THIS SURVEY IS ANONYMOUS.
The record of your survey responses does not contain any identifying information about you, unless a specific survey question explicitly asked for it.
If you used an identifying access code to access this survey, please rest assured that this code will not be stored together with your responses. It is managed in a separate database and will only be updated to indicate whether you did (or did not) complete this survey. There is no way of matching identification access codes with survey responses.

PRIVACY POLICY
INVITATION TO PARTICIPATE IN RESEARCH INFORMATION - CONSENT FORM
You are invited to participate in a scientific research entitled **"Stakeholder Perspectives on Real-World Data and Real-World Evidence for Health Technology Assessment"** as part of a PhD thesis with the scientific supervisor Assistant Professor Konstantinos Athanasakis of the Department of Public Health Policy of the School of Public Health of the University of West Attica.
The research was approved by the Research Ethics Committee of the University of West Attica (No. Prot 24994 - 28/03/2024).

Purpose of the Study: This survey aims to investigate the role of real-world data (RWD) and real-world evidence (RWE) in the health technology assessment process. The primary aim of this research is to conduct a qualitative exploration of international stakeholders' perspectives on the utilization of real-world data (RWD) and real-world evidence (RWE) in the context of health technology assessment (HTA). The study seeks to comprehensively understand the diverse viewpoints of various stakeholders, ranging from academia to industry, regulatory agencies, and patients. Your participation by completing this questionnaire is vital to gaining insights that can inform advancements in this field. You have been invited to take part in this research because there is a need to explore stakeholders' perspectives on real-world data in the health technology assessment process at an international level.

Voluntary Participation: Participation in this survey is entirely voluntary. You may choose to withdraw at any point without consequence, and without providing any reason. Your decision to participate or not will not impact your current or future relationship with the organization conducting the survey. If you participate in the survey, you will be asked to answer 21 questions.

Confidentiality and Privacy: Your privacy is of utmost importance. All information provided will be treated with strict confidentiality. Your responses will be anonymized, ensuring that no personally identifiable information is disclosed or shared with third parties.

ensure your privacy, your data will be anonymized/de-identified/encrypted via the LimeSurvey platform. Processing of your data will be based on your explicit consent, which you provide to us through this form. Your anonymity is guaranteed throughout the duration of the research and when publishing the results in conferences, scientific journals, etc.
The data collected will be used exclusively for research purposes. It will aid in analyzing trends, identifying areas for improvement, and contributing to the body of knowledge in health technology assessment. The data will not be retained in a format that permits the identification of individuals for a duration longer than necessary for the fulfillment of the processing purposes of this study – all data will be stored for maximum 3 years and after this period will be deleted. In particular, data will be collected and stored within a database on the LimeSurvey platform. It will be retained for the shortest period necessary to fulfill the study's objectives. An anonymized version of the responses will be maintained by the researcher for a maximum of 3 years, both within the database and on the researcher's personal computer as a backup. Upon reaching the end of the 3-year retention period or earlier, all data stored in both the database and on the researcher's personal computer will be securely deleted in compliance with GDPR requirements. Deletion procedures will ensure the permanent removal of all information, including backups and archived copies. Under GDPR regulations, you have the right to request the deletion of their data at any point. Upon receiving such requests, all your data will be promptly and securely deleted from the system, including from the researcher's personal computer. Additionally, you have the right to request access to their personal data held in the database and to request corrections or updates if necessary. Access to the database for study purposes will be restricted solely to authorized members of the research team.

Survey Distribution: This questionnaire is disseminated through LimeSurvey Cloud service to each participant, adhering to General Data Protection Regulation (GDPR) standards. Your participation implies consent for secure data handling.

Benefits, risks, and payments: Your involvement in the survey carries no discernible risks or financial obligations on your part. There is a risk that some of the questions may cause you some emotional upset / you may feel a little uncomfortable reading some of the questions.
Also, there are no specific benefits of participating in this survey. The benefit we seek is to gain more knowledge about the topic of the research so that we can formulate better policies on health technology assessment.
Importantly, there is no fee associated with your survey participation, and you will not bear any associated costs.

Contact Information: If you have any questions or concerns about the survey, you can contact Mr. **Konstantinos Zisis**, PhD Candidate at **kzisis@uniwa.gr**.

Complaints: For any complaint regarding the conduct of research, you may contact the Research Ethics Committee of the University of Western Attica (ethics@uniwa.gr). For any complaint regarding the management of your personal data, you may also contact the Data Protection Officer of the University of Western Attica, Mr Ioannis Agiopetritis (agiop@uniwa.gr). If your problem is not resolved, you can contact the Data Protection Authority by completing the form available on its website (complaints@dpa.gr).
Thank you for your valuable contribution to advancing knowledge in health technology assessment.
Konstantinos Zisis
Research Associate

CONSENT FORM
If you click on the "I agree" box, it means that:
- You have read all the above information
- You have read and understood all of the above information and have freely agreed to participate.
- You have read and understood all of the above information and agree to be bound by the terms and conditions of this Agreement.

☐ I agree

Next

Made in LimeSurvey

...

Section 1: Demographics

In this section, we would like to ask you a set of questions that provide essential background information about you. Demographic information helps us analyze our survey results in relation to specific participant attributes.It allows us to explore potential variations in responses based on different characteristics, providing a richer understanding of the survey data.

Question 1

Please specify your country of residence.

Question 2

What is your role within the healthcare system? Please provide a brief comment to describe your job title.

Comment only when you choose an answer.

- ☐ **Regulator/Policymaker**

- [ ] Pharmaceutical Industry Representative

- [ ] Patient Advocate

- [ ] Researcher/Academic

- [ ] Other

Question 3

How would you characterize your depth of expertise in Real-World Data (RWD)/Real-World Evidence (RWE) and the Health Technology Assessment (HTA) process, reflecting on your accumulated experience in these domains?

Choose one of the following answers

Please choose...

Question 4

How many years of experience do you have in your current field?

≤ 1 year

≥ 1 year to ≤ 3 years

≥ 3 years to ≤ 5 years

≥ 5 years

No answer

Previous

Next

Made in LimeSurvey

•••

This survey is currently not active. You will not be able to save your responses.

Section 2: Perceptions of Real-World Data for HTA

In the section titled "Perceptions of Real-World Data for HTA," we are aiming to gather insights from respondents about their views, opinions, and beliefs regarding the use of real-world data (RWD) and real-world evidence (RWE) in Health Technology Assessment (HTA). This section aims to explore how stakeholders perceive the value, challenges, and potential of using real-world data in the evaluation of health interventions.

Question 5

How important do you believe Real-World Data (RWD) is for Health Technology Assessment (HTA) in your country?

Choose one of the following answers

- Very Important
- Important
- Neutral
- Not Important
- Not Sure

Question 6

If your answer in question 5 was at least important, please indicate briefly the main reason of importance for including RWD and RWE in HTA.

Question 7

In your opinion, which health technologies and therapeutic areas/diseases do you believe RWD/RWE would be most applicable for within the framework of Health Technology Assessment (HTA)?

Rate your responses, by indicating X to each section that apply.

	Medicines	Vaccines	Medical devices	Tests/in-vitro tests	Digital technologies	Programs/procedures/interventions
Oncology						
Cardiovascular						
Diabetes						

	Medicines	Vaccines	Medical devices	Tests/in-vitro tests	Digital technologies	[illegible]cedures/interventions
Neurological Disorders						
Mental Health						
Infectious Diseases						
Autoimmune Diseases						
Rare Diseases						
Respiratory Diseases						
Renal Diseases						

Question 8

What types of Real-World Data are considered optimal and commonly sought for Health Technology Assessment (HTA) evaluations in your country?

Kindly choose one or more responses, by selecting 1 (not optimal) to 5 (very optimal).

	1	2	3	4	5	No answer
Patient/Disease Registries	○	○	○	○	○	○

- Electronic Health Records/Laboratory Data
- Patient Surveys/Diaries/Quality of Life Trackers
- Insurance/Claims/Biling Data
- Patient-Reported Outcomes/Wearable Device Data
- Healthcare Analytics Data
- Social Media
- Molecular Profiling Data
- Pharmacoepidemiology and Drug Safety Data
- Umbrella Studies and RCTs designed with RWE

Question 9

In your opinion, are there notable differences in the acceptance and utilization of RWD and RWE across different regions or countries?

If so, could you share some insights to support your perspective within the comment section?

Choose one of the following answers

- Yes, many significant differences
- Yes, notable differences

○ No, there no differences

○ No answer

Please enter your comment here:

Question 10

To what extent does your organization currently utilize/accept RWD and RWE in the HTA process?

Your explanation of the rationale behind your response would be greatly valued.

Choose one of the following answers

○ Extensively

○ Moderately

○ Minimally

○ Definitely not

○ Not sure/Don't know

○ No answer

Please enter your comment here:

Previous

Next

Made in LimeSurvey

Section 3. Barriers and challenges of RWD/RWE inclusion within HTA process

This section aims to gather insights from survey respondents about the barriers and challenges associated with incorporating Real-World Data (RWD) and Real-World Evidence (RWE) in the Health Technology Assessment (HTA) process. This section intends to explore both the potential barriers that might hinder the effective utilization of RWD/RWE as well as the challenges that might arise from their inclusion.

Barriers refer to the obstacles, difficulties, or limitations that might impede the smooth integration of RWD/RWE into the HTA process. Respondents will be asked to identify and explain any challenges they perceive when it comes to incorporating real-world data and evidence in the evaluation of health interventions.

Challenges in this context refer to the difficulties, obstacles, or problems that stakeholders perceive when attempting to include RWD and RWE in the HTA process. These challenges can arise from various aspects, such as data collection, data quality, methodology, stakeholder collaboration, and regulatory considerations.

Question 11

What are the main challenges you encounter regarding the quality and reliability of RWD in HTA?

You can select more than one answer and you can comment to supplement your answer.

- [ ] Low data quality

- [ ] Confounding biases

- [ ] Incomplete data

- [ ] Data protection and confidentiality concerns

- [ ] Other (please specify)

Question 12

Do you see fragmentation and lack of collaboration between stakeholders as significant barriers to the use of RWD in HTA?

Choose one of the following answers

- Yes, fragmentation and lack of collaboration are significant barriers
- No, I do not see them as significant barriers
- Somewhat, but there are ways to mitigate these challenges
- It depends on the context and specific stakeholders involved

impact

No answer

Question 13

What option do you suggest to address the barriers and challenges related to the use of RWD/RWE and health technology assessment (HTA) evaluations?

Kindly choose one or more responses. You can also comment to supplement your answer.

Comment only when you choose an answer.

- [ ] Data Quality, Harmonization, and Standardization

- [ ] Data Governance and Privacy

- [ ] Patient Engagement

- [ ] Collaboration and Stakeholder Engagement

- [ ] Transparency and Reproducibility

- [ ] Educational & Training Initiatives

- [ ] Streamline methodological procedures

- [ ] Infrastructure investments

- [ ] Other recommendation (please specify)

Question 14

How significant are the methodological challenges when utilizing real-world data and evidence in HTA?

Please comment to describe them briefly, if applicable.

Choose one of the following answers

- ○ Very significant
- ○ Significant
- ○ Moderate
- ○ Minor
- ○ Not significant
- ○ No answer

Please enter your comment here:

Question 15

To what extent does your organization possess the necessary expertise to effectively interpret and utilize real-world evidence (RWE) in decision-making processes?

Please indicate if you have any comments you'd like to add to supplement your answer with additional details.

Choose one of the following answers

- ○ Insufficient expertise
- ○ Moderate expertise
- ○ Sufficient expertise
- ○ High expertise
- ○ No answer

Please enter your comment here:

Question 16

acceptance of RWD and RWE in HTA that you would like to highlight?

Previous

Next

Made in LimeSurvey

...

Section 4: Future Outlook and Opportunities

This section aims to gather insights from respondents regarding their views on the potential developments, benefits, and opportunities related to the integration of Real-World Data (RWD) and Real-World Evidence (RWE) in the Health Technology Assessment (HTA) process. Here's a breakdown:

Section 4: Future Outlook and Opportunities

4.1. **Future Integration of RWD/RWE in HTA:** This part seeks to understand respondents' opinions on the likelihood of RWD/RWE becoming a more integral part of the HTA process in the future. By asking this question, you aim to gauge respondents' perceptions about the trajectory of RWD/RWE adoption in HTA.

4.2. **Potential Benefits and Opportunities:** In this section, respondents are encouraged to share their insights on the potential advantages and opportunities that could arise from incorporating RWD/RWE in HTA. Respondents may consider factors like improved decision-making, more accurate assessments, or faster evidence generation.

4.3. **Stakeholders' Roles in Promoting RWD/RWE Utilization:** Here, you're asking respondents to identify which stakeholders they believe should play a leading role in promoting the use of RWD/RWE in HTA. This question can shed light on how different stakeholders perceive their roles in shaping the future of RWD/RWE integration.

Question 17

How likely do you think it is that Real-World Data (RWD) will become a more integral part of Health Technology Assessment (HTA) in the future?

Choose one of the following answers

- ○ Very Likely
- ○ Somewhat likely
- ○ Neither likely nor unlikely
- ○ Somewhat unlikely
- ○ Very unlikely
- ○ Unsure / Don't know / Can't say
- ○ No answer

Please enter your comment here:

Question 18

What do you see as the main opportunities for using RWD and RWE in HTA?

Please rate from 1 to 5, with 1 representing the least feasible opportunity and 5 representing the most feasible opportunity.

Improved Decision-Making in HTA	○	○	○	○	○	○
Enhanced Efficiency in healthcare decision-making	○	○	○	○	○	○
Expanded Evidence Base for HTA assessments	○	○	○	○	○	○
Incorporation of Patient Perspectives	○	○	○	○	○	○
Facilitation of Comparative Effectiveness Research providing valuable insights into treatment efficacy and comparative outcomes	○	○	○	○	○	○
Identification of Unmet Needs and gaps in current treatment strategies	○	○	○	○	○	○
Support for Personalized Medicine in specific patient subpopulations	○	○	○	○	○	○
Long-Term Monitoring of Treatment Effects beyond the controlled settings of clinical trials	○	○	○	○	○	○
Facilitation of Health Technology Adoption by providing evidence of their real-world effectiveness and value	○	○	○	○	○	○
Support for Value-Based Healthcare	○	○	○	○	○	○

Question 19

enhance the integration of RWD and RWE into HTA processes? Please review the below list and rate from 1 to 5, with 1 representing the least necessary effort and 5 representing the most necessary effort.

	1	2	3	4	5	No answer
Interdisciplinary Collaboration between healthcare professionals, researchers, policymakers, and industry stakeholders						
Data Sharing Initiatives among stakeholders						
Standardization of Data						
Capacity Building and Training with training programs and capacity-building initiatives						
Public-Private Partnerships						
Patient Engagement with patient perspectives and preferences are incorporated into the use of RWD and RWE						
Harmonization of Methodologies and standards for conducting RWD and RWE studies						
International Collaboration between countries						

Collaboration between regulatory agencies and HTA bodies to align regulatory and HTA requirements for the use of RWD and RWE	○	○	○	○	○	○
Collaborative mechanisms for continuous evaluation and feedback among stakeholders involved in RWD and RWE integration into HTA processes	○	○	○	○	○	○

Question 20

What policy and regulatory measures do you believe are essential to support the effective integration of RWD and RWE in HTA?

Previous

Next

Made in LimeSurvey

Section 5: Additional Comments

The "Additional Comments" section of your survey is an open-ended space where respondents can provide any further thoughts, insights, concerns, or suggestions that may not have been covered in the previous sections.

Question 21

Is there anything else you would like to add regarding Real-World Data (RWD) and its role in Health Technology Assessment (HTA)?

Previous

Submit

Made in LimeSurvey
